# Cellular Interplay as a Consequence of Inflammatory Signals Leading to Liver Fibrosis Development

**DOI:** 10.3390/cells9020461

**Published:** 2020-02-18

**Authors:** Simona-Rebeca Ignat, Sorina Dinescu, Anca Hermenean, Marieta Costache

**Affiliations:** 1 Department of Biochemistry and Molecular Biology, University of Bucharest, 050095 Bucharest, Romania; simona.ignat@unibuc.ro (S.-R.I.); marieta.costache@bio.unibuc.ro (M.C.); 2 Research Institute of the University of Bucharest, 050663 Bucharest, Romania; 3 Institute of Life Sciences, Vasile Goldis Western University of Arad, 310414 Arad, Romania; anca.hermenean@gmail.com

**Keywords:** liver inflammation, liver fibrosis, sterile inflammation, inflammasome, DAMPs

## Abstract

Inflammation has been known to be an important driver of fibrogenesis in the liver and onset of hepatic fibrosis. It starts off as a process meant to protect the liver from further damage, but it can become the main promoter of liver fibrosis. There are many inflammation-related pathways activated during liver fibrosis that lead to hepatic stellate cells (HSCs) activation and collagen-deposition in the liver. Such events are mostly modulated upstream of HSCs and involve signals from hepatocytes and innate immune cells. One particular event is represented by cell death during liver injury that generates multiple inflammatory signals that further trigger sterile inflammation and enhancement of inflammatory response. The assembly of inflammasome that responds to danger-associated molecular patterns (DAMPs) stimulates the release of pro-inflammatory cytokines and at the same time, initiates programmed cell death called pyroptosis. This review focuses on cellular and molecular mechanisms responsible for initiation and progress of inflammation in the liver.

## 1. Introduction

Liver fibrosis affects liver structure with extracellular matrix (ECM) deposition and scar tissue formation, an important hallmark. Among the causes leading to liver fibrosis are viral hepatitis (B, C), excessive alcohol consumption, non-alcoholic steatohepatitis (NASH), metabolic diseases, autoimmune diseases, congenital syndromes. From all types of liver injury, liver fibrosis is a condition that could be reversed and liver integrity reestablished [1]. However, if the fibrous tissue accumulation affects too much liver function, liver fibrosis can turn into cirrhosis or hepatocellular carcinoma, both irreversible stages of liver disease. For this reason, it is important to know the events that lead to liver fibrosis onset, with special focus on one of the key cellular players in liver fibrosis, hepatic stellate cells (HSCs), which are activated by all the inflammation-related molecular changes in the liver [2,3].

**Figure 1:**
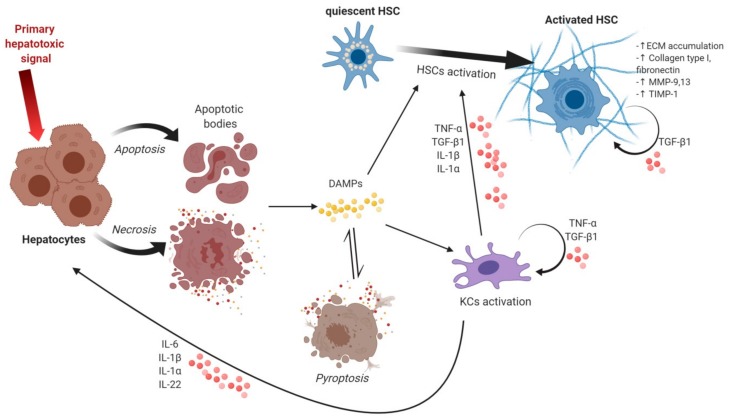
Events leading to hepatic stellate cells (HSCs) activation and accumulation of extracellular matrix. The hepatotoxic signal is the first to induce cellular death in hepatocytes by either apoptosis or necrosis, both types of death being followed by danger-associated molecular patterns (DAMPs) release. DAMPs can act on Kupffer cells (KCs) by activating them and trigger inflammatory cytokines and chemokines release that can act on hepatocytes, HSCs and on themselves. DAMPs can also be involved in promoting HSCs activation characterized by an increase in extracellular matrix (ECM) accumulation, collagen type I, fibronectin, metalloproteinase (MMP-9, 13), tissue inhibitor of metalloproteinases (TIMP-1), all fibrogenic markers. Pyroptosis is induced by DAMPs and triggers a self-perpetuating loop as more DAMPs are released following pyroptotic death. Figure created with Biorender.com.

Inflammation represents an important factor in chronic or acute liver disorder progress. It starts off as a process meant to protect the liver from further damage, but it can become the main promoter of liver fibrosis [3,4,5]. A series of events are set in motion by inflammation leading up to HSCs activation and collagen-deposition in the liver [5]. Such events are modulated upstream of HSCs and involve signals from hepatocytes and innate immune cells [6]. Almost every change that occurs in liver fibrosis affects the behavior of HSCs, supporting their transdifferentiation towards a myofibroblastic phenotype. In the normal liver, HSCs make up to 15% of the total liver cells and are located in the space of Disse. Following their activation, the quiescent vitamin A-storing HSCs turn into highly proliferative, fibrogenic and contractile myofibroblasts responsible for altered ECM-deposition (Figure 1) [5,7,8,9,10].

## 2. Events Triggering Hepatocyte Death, Release of DAMPs and Inflammatory Signals

Hepatocytes are parenchymal cells of the liver and are targeted by many hepatotoxic agents that may induce their death [6,7]. These hepatotoxic agents could be viruses, bacteria, bile acids, alcohol metabolites, etc. [11,12] that act as extracellular or intracellular cell death signals [4,13]. Most known extrinsic signals are members of the tumor necrosis factor (TNF) protein superfamily such as TNF-α, FASL, TNF-related apoptosis-inducing ligand (TRAIL) [14,15]. Of note is that TNF-α is massively produced when hematopoietic cells are exposed to bacteria or lipopolysaccharide (LPS) [16]. TNF-α is intercepted by death receptors such as ligand-bound TNR receptor 1 (TNFR1) [13,17] and this interaction is followed by reactive oxygen species (ROS) production and NF-κB activation [18,19]. Cell death can also follow intrinsic pathways triggered by accumulation of saturated fatty acids or response to redox stress [4,20,21]. Especially in NASH, lipotoxicity induces the endoplasmic reticulum (ER) stress response and the unfolded protein response (UPR). In mild ER stress conditions, homeostasis can be re-established [22], however, prolonged ER stress causes cell death by apoptosis via Bcl-2 family proteins [23,24].

Apoptosis is not the only type of cell death hepatocytes can follow, there are multiple other types of cell death (necrosis, necroptosis, pyroptosis) that can occur in the liver with different consequences on liver inflammation and fibrogenesis [25]. Necrosis is a type of cell death that occurs in hepatocytes, but unlike apoptosis, it generates more inflammatory signals. It is caused by mitochondrial deterioration, loss of membrane potential and ATP depletion that results in a process called oncosis characterized by cell swelling, coagulation of the cytoplasm and plasma membrane rupture followed by the release in the extracellular environment of the cellular content [25,26]. Necrosis is known as an unprogrammed cell death, but there are some forms of necrosis which are regulated, such as necroptosis. This type of cell death is regulated via death receptors (DRs) signaling such as pattern-recognition receptors (PRRs) or intracellular sensors [27,28]. Necroptosis follows the same upstream signaling pathway as apoptosis, but upon caspase-8 loss, it initiates necrosome assembly that results in cell death by cellular swelling and plasma membrane rupture with subsequent release of the cellular content [29,30].

Hepatocytes death further impacts HSCs activation in other ways as well. Their apoptotic bodies are engulfed by HSCs and it triggers the upregulation of transforming growth factor-β1 (TGF-β1) and procollagen 1 (I) and activation of reduced nicotinamide adenine dinucleotide phosphate (NADPH) oxidase which leads to superoxide production [31]. Moreover, dying hepatocytes release damage-associated molecular patterns (DAMPs) that act not only on HSCs directly by stimulating their activation, but also indirectly by inducing sterile inflammation on other cell types responsible for further hepatocyte damage [32].

DAMPs or alarmins are endogenous factors which are involved in various cellular functions in the healthy cells, but during cell death they are released in the extracellular medium and can induce sterile inflammation [26]. To date, there are multiple DAMPs identified that are usually intercepted by PRRs such as toll-like receptors (TLRs), cytosolic nucleotide-binding domain and leucine-rich repeat containing receptors (NOD-like receptors, NLRs) or C-type receptors (CLRs) [33]. This in turn stimulates the production of cytokines and activates the inflammatory response [34], along with the assembly of the inflammasome.

A partial list of DAMPs is presented in Table 1 with several factors very diverse, but all involved in sterile inflammation stimulation. One of the important sources of DAMPs is the mitochondria that can release mitochondrial DNA [33,34,35,36], formyl peptides [26,37], ATP and UTP [38,39,40]. MtDNA from damaged hepatocytes triggers TLR9, inflammasome and cyclic GMP-AMP synthase-stimulator of interferon genes (cGAS-STING) [41]. ATP is secreted through pannexin 1 channels from dying hepatocytes [42]. The ATP is intercepted by members of the P2 receptors family (P2RX7, P2Y2) and causes inflammasome activation, production of IL-1β and neutrophils infiltration [40,43]. In high concentrations, extracellular ATP initiates pore formation that can lead to cell death [44,45].

High mobility group box 1 (HMGB1) is a non-histone chromatin-binding protein released during cell death or by different immune cells as a response to other DAMPs [46,47,48,49]. It can be intercepted by some receptors from the TLRs family (TLR2, 4 and 9) and RAGE. Both receptor types respond to HMGB1 by activating intracellular signals such as nuclear factor (NF)-κB and the mitogen-activated protein kinase (MAPK) pathway [50]. HMGB1 can act in complex with other proinflammatory factors (single-stranded DNA, LPS, IL-1β and nucleosomes) to initiate inflammation [51]. On HSCs, recombinant HMGB1 promotes α-smooth muscle actin (α-SMA) expression and inhibits metalloproteinase-2 (MMP-2) activity [52]. Suppression of HMGB1 expression is correlated with downregulation of α-SMA and collagen (types I and III) in HSCs [53]. When acting along with TGF-β, HMGB1 stimulates the expression of more profibrogenic factors (collagen α2 and α-SMA) [54].

Uric acid is able to act as DAMP when found in a crystallization form after exposure to the extracellular environment [33,34,35,36,37,38,39,40,41,42,43,44,45,46,47,48,49,50,51,52,53,54,55]. This form of uric acid stimulates inflammation without binding to an extracellular receptor and can cause phagosome rupture and activation of cytosolic proteases [35]. In the case of cellular injury, histones are also released in the extracellular medium and act as DAMPs. They bind with TLR2/4 and induce sterile inflammation with NLRP3 inflammasome formation [36,56,57,58]. Nuclear DNA released from acetaminophen-exposed hepatocytes interacts with TLR9 and additionally to its NLRP3 inflammasome activation [59], nDNA also stimulates HSCs transdifferentiation to myofibroblasts [60]. Free fatty acids (FFA) are other proposed DAMPs which can activate TLR4 by fentuin-A modulation. Studies on mice show a link between fentuin-A expression and TLR4-mediated inflammatory signaling in adipose tissue. However, there is evidence fentuin-A might act in the liver as well since its levels are elevated in non-alcoholic fatty liver disease (NAFLD) [22]. Other DAMPs worth mentioning are CRT and ERp57 that bind to the CD91 receptor and represent ER chaperons. They are released in ER stress via the Golgi secretory pathway [61,62].

Recent studies have identified new DAMPs associated with liver injury. Peroxiredoxin-1 (Prdx1) is one of them and it is recognized by TLR2/4 and further initiates NLRP3 inflammasome [63,64,65]. Prdx1 is involved in the production of pro-inflammatory cytokines such as IL-1β, IL-6 and TNF-α by acting on NF-κB and NLRP3 inflammasome pathways. In a study by He et al., [64] levels of circulating Prdx1 in mice with acute liver injury (ALI) are increased, as well as for patients with ALI.

Some DAMPs may present an anti-inflammatory effect when they interact with other receptors. Such is the case for HMGB1 and HSPs when binding to CD24 or sialic acid-binding Ig-like lectin 10 (SIGLEC10) [66]. Additionally, IL-33 which is a transcription factor in healthy cells, during inflammation, is thought to inhibit inflammatory reactions [67,68]. Heat-shock proteins (HSPA1A, HSPB1) represent other potential DAMPs that interact with TLR2/4 and are involved in the hepatic response to ischemia reperfusion (IR). Their role might also be anti-inflammatory and act as intracellular chaperons [69,70].

## 3. Activation of KCs and Inflammatory Signaling

### 3.1. Inflammatory Signals from Kupffer Cells and Other Inflammation-Related Cells

Kupffer cells (KCs) are specialized mesenchymal macrophages that are part of the reticuloendothelial system (RES) and make up around 30% of hepatic sinusoidal cells. These types of cells have an important role in the defense against infections derived from the gastrointestinal tract. KCs can be activated by a number of phagocytosable particles and soluble substances by binding to specific PRRs such as TLRs, mannose receptors and NOD-like receptors (NLRs) [71]. For instance, KCs can respond to exposure to LPS by binding to TLR4 which leads to production of inflammatory cytokines (TNF-α, IL-1β, IL-6, IL-12, IL-18, etc.) and other chemokines [72], most of them being actively involved in HSCs activation (Table 2) [5,73,74,75]. Other macrophages recruited to the liver in the case of inflammation, are also involved in secretion of pro- and anti-inflammatory cytokines, depending on their type (proinflammatory M1 or immunosuppressive M2, respectively). M1 macrophages release TNF-α, IL-1 and IL-6 when stimulated by interferon- γ (IFN-γ), LPS, TNF-α, or IL-17, while M2 macrophages produce IL-10, TGF-β, platelet-derived growth factor (PDGF) and epidermal growth factor (EGF) when stimulated by IL-4, IL-10, or IL-13 (Table 2) [76,77,78].

Another great source of inflammation in the liver is brought on by cells involved in innate immunity, such as Th17 cells, natural killer (NK) cells and mucosal-associated invariant T (MAIT) cells. Th17 cells are responsible for IL-17 production, a cytokine that stimulates liver fibrosis. NK cells are activated by the release of retinoic acid from transdifferentiating HSCs and they further attack activated HSCs and produce IFN-γ [5,14,79,80]. MAIT cells are innate-like T cell populations [81] that are found in the peri-biliary areas of portal tracts and secrete cytokines such as IL-17, granzyme B (Gr-B), IFN-γ and TNF-α. Activated MAIT cells induce a proinflammatory behavior of hepatic myofibroblasts by stimulation of IL-8 and IL-6 production [82].

TGF-β1 is a multifunctional cytokine and one of the key regulators and promoters of liver fibrosis stimulating HSCs transdifferentiation from quiescent cells to activated myofibroblasts [83]. TGF-β1 is synthetized by activated HSCs (aHSCs), KCs, hepatocytes and liver sinusoidal endothelial cells (LSECs) and exerts its role on liver cells via type I and II TGF-β receptors (TβRI and TβRII), as well as SMAD-mediated intracellular signals [7,84]. TGF-β1 mediates transdifferentiation of HSCs to myofibroblasts by upregulation of Notch pathway markers and α-SMA [83]. Additionally, it stimulates expression of collagen type I and other matrix-producing genes [85].

TNF-α is a pleiotropic cytokine produced mainly by KCs and other inflammatory-related cells that stimulates cell proliferation, inflammatory response and even cell death [86]. In aHSCs, TNF-α can induce a pro-survival signal by activating NF-κB, Bcl-XL and p21 [87]. Additionally, it stimulates IL-6 and IL-8 production [4,18]. As of cell death stimulation, TNF-α acts on receptor TNF-R1 that leads to interaction with TNF receptor-associated protein with death domain (TRADD) and forms a complex with TNF-α receptor-associated factor 2 (TRAF2), receptor-interacting kinase (RIP) and adapter molecule Fas-associated death domain (FADD) that further activate caspase-8 and induce apoptosis [88].

### 3.2. Proinflammatory Cytokines

Among all cytokines present in liver fibrosis, IL-17 and IL-1 families distinguish themselves as important proinflammatory cytokines. IL-17 acts by two different mechanisms to exert its profibrogenic effect. Firstly, IL-17 can induce production of IL-6, IL-1β, TNF-α and TGF-β1 in KCs and other macrophages that will further stimulate, indirectly, HSCs activation. Secondly, IL-17 can also act directly on HSCs, by promoting collagen type I deposition and an increase in fibrogenic markers expression such as α-SMA and TGF-β1 [78,89].

IL-1 cytokine has a pro-fibrogenic effect on HSCs by upregulation of MMP-9, MMP-13 and tissue inhibitor of metalloproteinases (TIMP-1) [90]. IL-1 family comprises of 11 members and some of the best known and most studied are IL-1α, IL-1β and IL-18 [91]. IL-1α and IL-1β act synergistically in promoting development of liver fibrosis, as shown in a study that blocking the expression of either of them *in vivo* was sufficient to protect mice from developing steatohepatitis [92]. IL-1β is mostly produced by KCs and has pro-inflammatory effects in the development of NASH by inhibiting peroxisome proliferator-activated receptor-α (PPAR-α) and enhancing the TNF-α role in cell death stimulation [93,94]. Depending on the type of liver fibrosis driving infection, IL-1β can be one of the main factors underlying inflammation as in the case of chronic HBV infection, but not in chronic HCV infection [95]. In rat HSCs, IL-1β stimulates proliferation via IL-1 type I receptor (IL-1R1), JNK and AP-1 pathway [96].

### 3.3. Anti-Inflammatory Cytokines

IL-10 is involved in downregulating the pro-inflammatory processes in liver fibrosis [7]. *In vivo* studies showed that IL-10 inhibits the expression of aHSCs markers demonstrating its anti-fibrotic effects [97,98]. In a more recent study, IL-10 was shown to promote cellular death of aHSCs by senescence and upregulation of p53 and p21 expression [98]. Another anti-inflammatory cytokine is IL-22 that belongs to the IL-10 family and it is produced by innate immune system cells [99,100,101]. IL-22 acts upon hepatocytes via a transmembrane receptor complex with two subunits (IL-22 receptor 1 and IL-10 receptor 2) [102]. It induces upregulation of anti-apoptotic and mitogenic proteins, promoting hepatocyte survival in the context of liver injury [103,104,105]. IL-22 can also activate signal transducer and activator of transcription 3 (STAT3) signaling in HSCs and induce their death by senescence, contributing to resolution of liver fibrosis [105]. At the same time, IL-22 was shown to inactivate HSCs by downregulation of the TGF-β1/Notch signaling pathway in HSCs [106].

IL-6 is a pleiotropic cytokine, mostly produced by KCs that can act in acute and chronic inflammation with a different type of action, pro- or anti-inflammatory. It can regulate NF-κB and Ras-MAPK pathways to inhibit apoptosis and stimulate regeneration in hepatocytes [107,108]. IL-6 is a major activator of STAT3 involved in the development of many liver diseases and especially hepatocellular carcinoma [109]. Additionally, IL-6/STAT3 signaling has been correlated with liver fibrosis and HSC activation [110,111].

## 4. Sterile Inflammation and Inflammasome Complex

**Figure 2:**
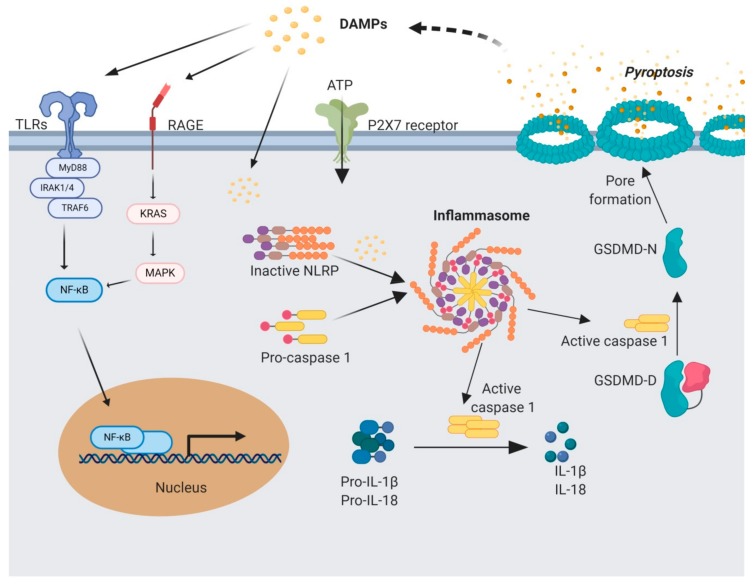
Mediators involved in sterile inflammation signaling and pyroptosis. Sterile inflammation is triggered by a first signal triggered by DAMPs that can act on receptors such as toll-like receptors (TLRs), RAGE and PRX7. Activating signaling by TLRs initiates a cascade that translocates NF-κB in the nucleus and stimulates pro-IL-1β and pro-IL-18 expression. A second signal stimulates inflammasome assembly from NOD-like Receptors (NLRs), caspase activation and recruitment domain (CARD) and pro-caspase 1 that is cleaved and activated within this complex. Activated caspase-1 further activates IL-1β and IL-18 and also releases the *N*-terminal of gasdermin, responsible for pore formation and initiation of pyroptosis. IL-1β, IL-18, along with DAMPs are released from the cell by means of the pores and initiates a self-perpetuating loop of sterile inflammation. Figure created with BioRender.com.

The inflammasome is a cytosolic multiprotein complex comprised of NLR, apoptosis-associated speck-like protein containing a CARD (ASC) and caspase 1/5 [112,113]. They are responsible for the cleavage and secretion of pro-IL1β and pro-IL18 [114] (Figure 2). There are two types of NLRs depending on the *N*-terminal domain, NOD-like receptor family with pyrin domain (NLRP) and NOD-like receptor family with caspase activation and recruitment domain (CARD) (NLRC) [115]. Depending on the type of receptor, there are several types of inflammasomes such as NLRP1, 2, 3, 6, 10, 12, NLRC4 and AIM2 that may act on different pathways, but they all activate caspase-1. So far, the activity of only NLRP1, 3, NLRC4 and AIM2 inflammasomes was identified in liver inflammation, a great number of studies being focused on the role of NLRP3 inflammasome [116]. Each type of inflammasome is activated by different signals and has different roles depending on the cell type involved [113]. NLRP1 inflammasome was shown to be activated by anthrax lethal toxin, bacterial muramyl dipeptide and infection with *Toxoplasma gondii* [117]. Additionally, NLRP1 can be activated by metabolic disturbances such as ATP-depletion [118,119]. NLRC4 inflammasome is thought to be mostly activated as a defensive mechanism against bacterial infection (*Salmonella, Legionella* and *Shigella*) [113]. However, other non-bacterial factors can induce NLRC4 activation as well, such as free fatty acids (FFA) shown in a recent study by Chen et al. [120]. The study was conducted on a NAFDL in vitro cell model based on HepG2 cells and THP-1 macrophages co-culture. The study showed that activation of NLRC4 inflammasome is TNF-α mediated and initiates pyroptosis with NLRC4 translocation in the mitochondria. AIM2 inflammasome responds to cytosolic double-stranded DNA (dsDNA) through its C-terminal DNA binding domain and *N*-terminal pyrin domain [121]. This type of inflammasome can be activated by dsDNA originated from both the host or other pathogens such as *Listeria monocytogenes, Schistosoma mansoni* [122], or viruses such as chronic hepatitis B virus [123]. Activation of AIM2 inflammasome triggers cell death by apoptosis. In the case of some bacterial infections, a possible crosstalk is possible between AIM2 and NLRP3 inflammasomes as it was recently hypothesized in a study by Chen et al. (2019) [120]. NLRP3 does not trigger apoptosis like the other NLRs, but it is mostly involved in regulating innate immune response as a sensor for tissue stress [124].

Regardless of its type, the inflammasome is a key modulator of the events triggering liver fibrosis, controlling the activation of proinflammatory caspase 1. In response to this activation and release of IL-1β and IL-18 cytokines, an abnormal wound healing and pro-regenerative signal is initiated, further leading to HSCs activation [6]. Recent studies have shown that NLRP3 activation is actually required for hepatic inflammation and fibrosis [125], although it is not clear yet if this inflammasome assembly directly leads to HSC activation or if it first creates the necessary inflammatory microenvironment that triggers itself HSC recruitment from the quiescent state and change in phenotype. What is more, NLRP3 overactivation *in vivo* long term resulted in increase of fibrogenesis-related markers and collagen secretion, without significant elevation of the inflammatory profile.

Although the general overview suggests that the inflammasome activation has a negative impact on cell fate by directly inducing pyroptosis, there is recent evidence on the dual role of inflammasomes in liver diseases [126]. On one hand, inflammasomes are needed for protection against danger signals, oxidative stress and pathogen infections, but on the other hand their overactivation favors the development of several liver pathologies. In addition, NLRP3 activation may lead also to abnormal wound healing events associated to fibrosis, in parallel to triggering pyroptosis. Nevertheless, NLRP3 inflammasome acts indirectly on the adaptative immune system by stimulating caspase-1 and IL-1β production, which favor Th17 cell differentiation and secretion of IL-17, resulting in continuous liver fibrosis state [127]. All these facts argue that the dual role of inflammasomes in liver fibrosis and might represent an opportunity to develop therapies targeting them. Up to now, these therapies were designed to act at either the level of caspase-1 (caspase inhibitors) or at the level of IL-1β signaling (IL-1β inhibitors) [128].

Liver fibrosis state was found to be regulated both directly and indirectly by inflammasomes [128]. The indirect pathway involves HSCs activation by IL-1β and IL-18 derived from KCs [129], while the direct inflammasome activation in HSCs was found when inducing TGF-β upregulation by treatment with uric acid crystals or derivatives [130,131].

The release of fully functional IL-1β and IL-18 cytokines depends on a two-step process. TLRs send the first signal that initiates the transcription of cytokines pro-IL-1β and pro-IL-18 which require post-transcriptional processing by caspase-1 cleavage to activate them. However, caspase-1, expressed as pro-caspase-1 also needs to be activated by assembly in the inflammasome complex. The second signal initiates the inflammasome formation and activated caspase-1 cleave and release IL-1β and IL-18 cytokines [132,133]. The second signal needed for inflammasome activation can be transmitted in multiple ways. Cytosolic Ca^2+^ from the release of mitochondrial reactive oxygen species (mROS) and mitochondrial DNA (mtDNA) can activate NLRP3 inflammasome [134]. G protein-coupled Ca^2+^-sensing receptors (CASR and GPRC6A) and Ca^2+^-permeable channels (transient potential melastatin-like 2 (TRPM2), TRPV2 and TRPM7) ensure NLRP3 activation [135,136,137]. Another stimulant can be endoplasmic reticulum (ER) stress modulated by thioredoxin-interacting protein (TXNIP) [138,139,140].

Caspase-1 activation also leads to the release from the cell of IL-1α and members of the caspase-1 secretome such as HMGB1 [141,142,143]. Their release in the extracellular medium stimulates the immune system as they are perceived as DAMPs, proving this as a self-perpetuating loop of inflammation [6,144].

Furthermore, activated caspase-1 can initiate canonical pyroptosis, a lytic form of programmed cell death characterized by cell swelling, cell lysis and the release of the cytoplasmic content [145,146]. It has evolved as a way of removing intracellular pathogens in a distinctive way compared to other forms of cell death [25]. There are additional caspases involved in the onset of noncanonical pyroptosis along with caspase-1 activated by the inflammasome, such as caspase-4, 5, 8, 11 and 12 [147,148,149]. The specific role of activated caspases is to cleave gasdermin-D (GSDMD) and the resulting *N*-terminal peptide fragment binds to the plasma membrane. This binding initiates pore formation that leads to membrane integrity loss and cellular content release followed by cellular death [148,150,151]. This type of cell death enhances the immune response by releasing triggers such as ATP, HMGB-1 and IL-1α, but also IL-1β and IL-18 inflammatory cytokines [148,152]. Pyroptosis is an important process involved in the development of different types of liver diseases, with various levels of expression in all the liver cell types. It occurs most commonly in macrophages and hepatocytes and can be triggered by DAMPs released by primary damaged hapatocytes [149]. In a study of Mirshafiee et al. [153] it was shown that KCs undergo pyroptosis as a result of direct exposure to rare earth oxides. Moreover, in another recent study, NLPR3 inflammasome in KCs was demonstrated to play an important role in the progress of NASH in an animal model [154]. Hepatocytes can also be stimulated to activate inflammasomes and follow pyroptotic death. In a study by Lebeaupin et al. [155], it was shown that ER stress in a mice model of steatohepatitis leads to NLRP3 inflammasome overexpression resulting in liver inflammation and hepatocytes undergoing pyroptosis. Another study by Heo et al. confirmed NLRP3 inflammasome overexpression and subsequent pyropotosis in hepatocytes after ethanol exposure in human and animal liver samples with alcoholic hepatitis [156].

As pyroptosis was demonstrated to be induced under certain conditions in KCs, hepatocytes and HSCs, further studies confirmed the relevance of this cell death in the development of different types of liver diseases [95,157,158,159]. Some of the most studied liver diseases are NAFLDs that could vary from steatosis to fibrosis after prolonged chronic inflammation in the liver. In NASH, NLRP3 inflammasome is activated, GSDM is cleaved and IL-1β and IL-18 are released after pyroptosis, molecules that contribute to the progress of the disease [149,160,161]. In alcoholic liver disease (ALD) development, the role of pyroptosis has been as well established in several studies [156,162]. Particularly, one study showed that NLRP3 deficiency in alcohol-fed mice protected the liver against inflammation and ameliorated liver damage and steatosis progress [157].

## 5. Conclusions

Liver fibrosis represents a reversible pathological condition, closely related to inflammation, which affects the hepatic structure and function by accumulation of excessive ECM. Under chronic stimuli, liver fibrosis can progress to irreversible stages of liver damage such as cirrhosis or hepatocellular carcinoma. The reversibility of liver fibrosis is influenced by the pro-fibrogenic signals and the microenvironment created by the interplay and coordination between hepatocytes, Kypffer cells and HSCs. Under the action of hepatotoxic agents, hepatocytes are the first cells to be affected by undergoing cell death, releasing danger signals. KCs and other inflammatory-related cells are also alerted by the presence of hepatotoxic agents in the liver and once activated, produce inflammatory cytokines that further act on all the other cell types to maintain the inflammatory state. Therefore, this cellular interplay highly affects the overall response to danger signals, in most cases, leading to HSCs activation and supporting their transdifferentiation towards a myofibroblastic phenotype, highly proliferative and responsible for matrix accumulation.

Sterile inflammation further promotes fibrogenesis and the inflammasome assembly is responsible for a self-perpetuating loop of an inflammatory state. Contradictory to the general belief that the inflammasome activation has a negative outcome favoring cell death by pyroptosis, actually inflammasomes display a dual role in liver diseases. On one hand, inflammasomes are needed for protection against danger signals, oxidative stress and pathogen infections, but on the other hand their overactivation may lead to abnormal wound healing events associated with fibrosis, in parallel to triggering pyroptosis.

There are still many unknown mechanisms of inflammasome signaling in liver fibrosis that will require further studies to shed light over the correlation between inflammasome types and their role in different types of liver injury. All this knowledge on the subject can open opportunities to develop therapies that target inflammasomes, to either inhibit inflammasome assembly, or act on the downstream signaling molecules.

## Figures and Tables

**Table 1:** List of DAMPs, receptors and their function in liver inflammation.

DAMPs	Receptor	Function
ATP, UTP	P2RX7, P2Y2, NLRP3	Purine metabolites
		NLRP3 inflammasome activation Neutrophils infiltration
Defensins	TLR4, CCR6	
Fatty acids	TLR4	Inflammatory signaling
HSPs (HSPA1A, HSPB1)	TLR2, TLR4, CD14, CD91	Intracellular chaperons Adjuvants
CRT, ERp57	CD91	ER chaperons
HMGB1	TLR4, RAGE, CD24/SIGLEC10	Multifunctional nuclear factor Profibrogenic effects Anti-inflammatory function (binding to RAGE, CD24/SIGLEC10)
IL-33	IL1RL1	Anti-inflammatory function
mtDNA	NLRP3, TLR9	NLRP3 inflammasome activation
nDNA	NLRP3, TLR9	NLRP3 inflammasome activation HSCs activation
*N*-formyl peptides	FPR1, TLR9	Mitochondrial polypeptides
S1P	S1PR	Anti-apoptotic stimulus
Uric acid	NLRP3	Purine catabolite Pro-inflammatory metabolite
Peroxiredoxin-1	TLR2, TLR4, NLRP3	NF-κB and NLRP3 inflammasome signaling
Histones	TLR2, TLR4	NLRP3 inflammasome activation

**Table 2:** Inflammatory cytokines involved in liver inflammation.

Inflammatory Signal	Producing Cells	Target Cells	Role
TNF-α	KCs, M1 macrophages, MAIT cells	Activated HSCs	Pro-survival of aHSCs
TGF-β1	aHSCs, KCs, hepatocytes, LSECs	HSCs, KCs	HSCs activation, upregulation of matrix-producing genes
IL-17	Th cells, MAIT cells	KCs, M1 macrophages	Indirectly HSCs activation, HSCs collagen type I deposition
IL-10	M2 macrophages	M2 macrophages, aHSCs	Downregulating pro-inflammatory processes, senescence of aHSCs
IL-6	KCs, M1 macrophages, myofibroblasts	Hepatocytes	Inhibition of apoptosis and regeneration stimulation of hepatocytes
IL-1β	KCs	HSCs, hepatocytes	Pro-inflammatory effect, acts together with Il-1α, HSCs proliferation
IL-1α	KCs	HSCs, hepatocytes	Pro-inflammatory effect, acts together with Il-1α
IL-22	KCs, innate immunity cells	Hepatocytes	Pro-survival signals on hepatocytes
